# A Channel Rejection Method for Attenuating Motion-Related Artifacts in EEG Recordings during Walking

**DOI:** 10.3389/fnins.2017.00225

**Published:** 2017-04-26

**Authors:** Anderson S. Oliveira, Bryan R. Schlink, W. David Hairston, Peter König, Daniel P. Ferris

**Affiliations:** ^1^Human Neuromechanics Laboratory, School of Kinesiology, University of MichiganAnn Arbor, MI, USA; ^2^Department of Materials and Production, Aalborg UniversityAalborg, Denmark; ^3^U.S. Army Research Laboratory, Aberdeen Proving GroundAberdeen, MD, USA; ^4^Institute of Cognitive Science, University of OsnabrückOsnabrück, Germany; ^5^Department of Neurophysiology and Pathophysiology, University Medical Center Hamburg-EppendorfHamburg, Germany

**Keywords:** EEG, artifacts, walking, locomotion, signal processing, mobile-brain imaging, ICA, EEG cleaning

## Abstract

Recording scalp electroencephalography (EEG) during human motion can introduce motion artifacts. Repetitive head movements can generate artifact patterns across scalp EEG sensors. There are many methods for identifying and rejecting bad channels and independent components from EEG datasets, but there is a lack of methods dedicated to evaluate specific intra-channel amplitude patterns for identifying motion-related artifacts. In this study, we proposed a template correlation rejection (TCR) as a novel method for identifying and rejecting EEG channels and independent components carrying motion-related artifacts. We recorded EEG data from 10 subjects during treadmill walking. The template correlation rejection method consists of creating templates of amplitude patterns and determining the fraction of total epochs presenting relevant correlation to the template. For EEG channels, the template correlation rejection removed channels presenting the majority of epochs (>75%) correlated to the template, and presenting pronounced amplitude in comparison to all recorded channels. For independent components, the template correlation rejection removed components presenting the majority of epochs correlated to the template. Evaluation of scalp maps and power spectra confirmed low neural content for the rejected components. We found that channels identified for rejection contained ~60% higher delta power, and had spectral properties locked to the gait phases. After rejecting the identified channels and running independent component analysis on the EEG datasets, the proposed method identified 4.3 ± 1.8 independent components (out of 198 ± 12) with substantive motion-related artifacts. These results indicate that template correlation rejection is an effective method for rejecting EEG channels contaminated with motion-related artifact during human locomotion.

## Introduction

Electroencephalographic (EEG) recordings have traditionally been limited to highly controlled conditions in order to avoid head motion and minimize artifacts that may be large in amplitude compared to the desired signals. In the last decade, advances in hardware and software improved the quality of the acquired signals and opened the venues for recordings in more dynamic conditions including whole-body motion (Gramann et al., 2010; Gwin et al., 2010; Reis et al., 2014; Seeber et al., 2015). A common method for analyzing EEG data is to apply independent component analysis (ICA) and blind source localization in order to define dipolar sources of electrocortical activity (Makeig et al., 1996, 2002; Gwin et al., 2011; Delorme et al., 2012). Finally, event-related spectral perturbation analysis (ERSPs) (Pfurtscheller and Lopes da Silva, 1999; Delorme et al., 2012) identifies synchronization and desynchronization of selected brain sources and/or clusters of sources in relation to task phases. For the motor system, neuronal synchronization and desynchronization are responsible for the modulation of movement control, being more relevant at the theta, alpha, beta and gamma bands (Gwin et al., 2011; Wagner et al., 2012, 2016; Seeber et al., 2014, 2015). The combination of these signal processing methods has been widely used to describe the participation of different cortical areas on the control of human walking (Gwin et al., 2011; Castermans et al., 2014; Ehinger et al., 2014; Seeber et al., 2014; Wagner et al., 2014; Bulea et al., 2015).

Recording EEG during natural human behavior poses specific challenges. Locomotion induces vertical head acceleration/deceleration in every step, and this periodic oscillation may be reflected in the EEG recordings due to cable sway and/or electrode movements on top of the head (Gwin et al., 2010; Reis et al., 2014; Kline et al., 2015; Oliveira et al., 2016b). Recent studies have shown some correlations between head acceleration and the EEG amplitude for some channels (Kline et al., 2015; Onikura et al., 2015). Moreover, changes in head displacement can influence event-related spectral perturbations in relevant frequency ranges up to 15 Hz, and gait-related artifacts might span up to high-gamma frequency range (>60 Hz, Castermans et al., 2014; Costa et al., 2016). There is an inherent problem with the analysis of brain dynamics related to locomotion: gait-related motion artifacts are likely to occur in a systematic pattern, locked to certain gait events, considering forward locomotion at steady speed. In other words, a specific amplitude pattern may be present in the majority of the gait cycles during certain phases of the gait cycle, such as initial heel strike. As a result, both relevant brain activity and artifacts could be tightly coupled throughout the data collection, regardless of the duration. Independent component analysis has shown to be good at identifying motion artifacts in scalp EEG data (Snyder et al., 2015; Oliveira et al., 2016a), but additional methods that can attenuate motion artifacts in scalp EEG could improve signal quality further, especially at faster walking speeds (Nathan and Contreras-Vidal, 2016).

Because of this time and phase-locked coupling between artifacts and neural motor control circuits, there are controversies on the validity of event-related spectral perturbation analysis reported by different research groups concerning brain dynamics of the modulation of locomotion (Gwin et al., 2011; Presacco et al., 2011; Petersen et al., 2012; Castermans et al., 2014). Specifically, it is often questionable whether changes in event-related spectral perturbation plots are a true neural oscillation or motion artifact (Castermans et al., 2014; Snyder et al., 2015) however, there is a lack of research directly addressing this topic. The influence of motion-related artifacts on EEG recordings acquired from freely moving humans is a major limitation for future studies regarding brain activity in real-world conditions (McDowell et al., 2013). Therefore, alternative solutions to reduce the influence of motion on EEG results are necessary.

**Figure 1:**
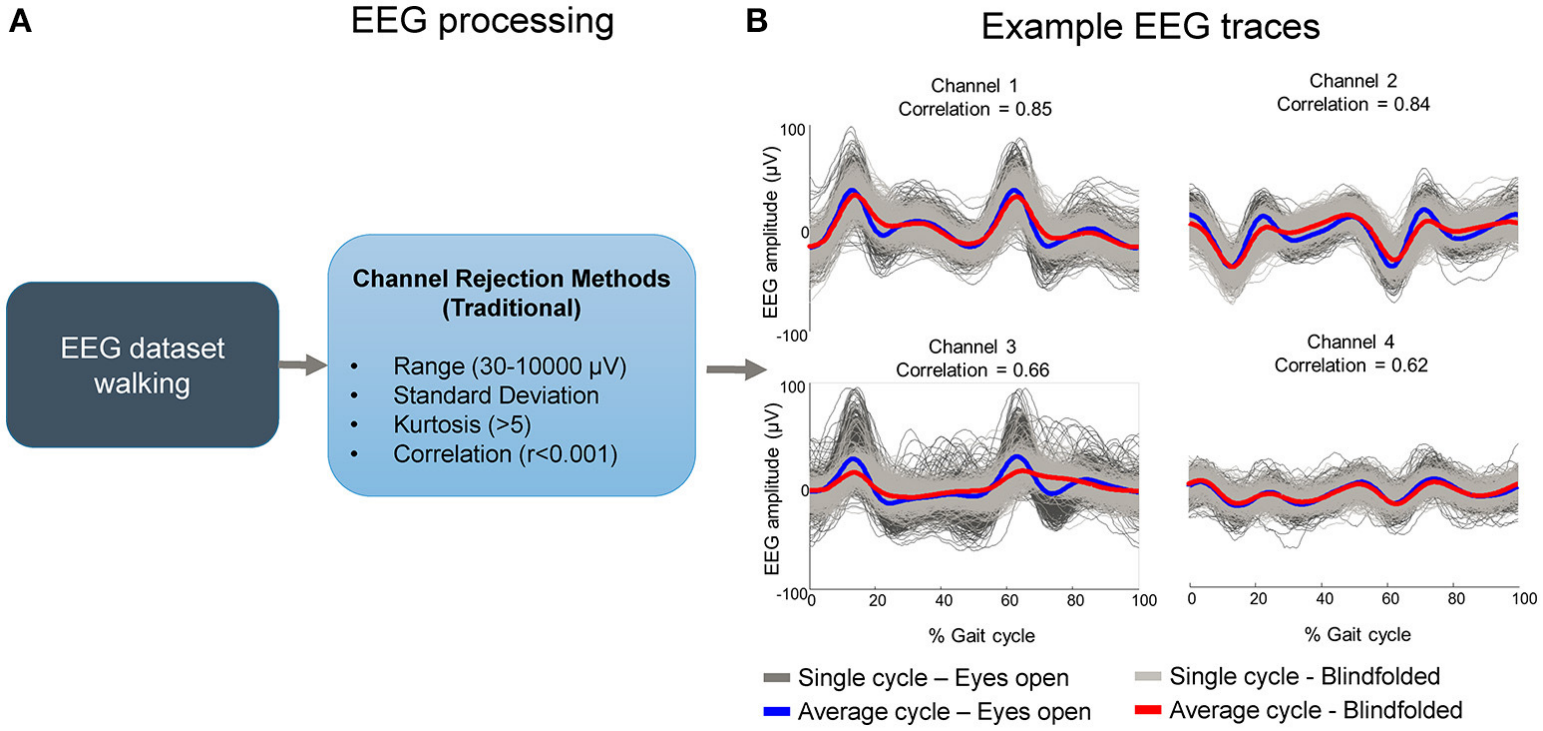
**Potential flaws in rejecting EEG channels containing motion-related artifacts using commonly accepted methods**. Bad channels are usually selected by using traditional methods such as range, standard deviation, kurtosis and correlation **(A)**. These methods may not be sensitive to channels carrying motion-related artifacts if the amplitude is not substantially high and/or if the correlation across channels is not sufficiently high. In such cases “clean” EEG datasets may contain channels carrying artifacts locked to gait events while walking with eyes open and blindfolded in the majority of the time series **(B)**.

The current methods for the selection of bad channels do not account for any event-locked pattern (Figure 1A). As a result, EEG channels carrying repetitive motion-related artifacts locked to some motion event may persist for further processing if the researcher cannot identify this channel by visual inspection or any other alternative method (Figure 1B). The goal of this study was to test a systematic method for identifying EEG channels dominated by motion-related artifact during human locomotion data collection. Specifically, we devised a template correlation rejection (TCR) method using pattern templates and the fraction of total epochs correlated to the template. This method can identify EEG channels affected by motion-related artifacts from cyclical motion, such as walking, as well as independent components carrying motion-related artifacts.

## Methods

### Participants

Ten healthy volunteers with no history of major lower limb injury and no known neurological or locomotor deficits completed this study (6 males and 4 female; age range, 21–36 years). All subjects provided written informed consent before the experiment. All procedures were approved by the University of Michigan Internal Review Board and complied with the standards defined in the Declaration of Helsinki.

### Experimental protocol

Subjects performed a finger tapping task and two walking tasks in randomized order. For the finger tapping task, subjects sat comfortably in a chair and performed 5 sets of rhythmic finger tapping for 60 s using their right thumb. The interval between sets was ~60 s. We included this finger tapping task in order to compute the amplitude range (in μV) of EEG signals recorded in a highly controlled seated condition and subsequently compare it to the amplitude acquired during walking. Compared to the foot region, that is placed in mirror symmetrically on the medial surface of both cortical hemispheres, the hand related part of motor cortex is positioned in both hemispheres closely below the skull (Penfield and Boldfrey, 1937; Liao et al., 2014). Thus, activity generated by finger movements should relay well to scalp electrodes.

For the walking task, subjects walked on an instrumented split belt treadmill (Bertec FIT, Bertec Inc. USA) at 1 m/s in two different conditions: (1) Eyes open, subjects walked normally avoiding anterior-posterior or lateral head movements; (2) Walking blindfolded, subjects closed their eyes while wearing a blindfold and walked avoiding anterior-posterior and lateral head movements. In addition, we turned off all lights in the laboratory environment in order to minimize the possibility of visual guidance. We chose to investigate subjects walking in these two conditions in order to induce changes in the walking pattern at a fixed speed. The lack of visual guidance induces a more cautious walking pattern (Nakamura, 1997), which may attenuate the influence of head acceleration/deceleration on our EEG recordings and highlight the effectiveness of the proposed method for identifying bad channels. For both conditions we attached the subjects to a safety harness, and they alternated five min walking with their eyes open and five min walking blindfolded until completing five blocks of each condition (total of 25 min walking with eyes open and 25 min walking blindfolded). There was a 1–2 min interval between each block.

### EEG recordings

EEG was recorded using a compact ActiveTwo amplifier and 256-channel active electrode array (BioSemi, Amsterdam, The Netherlands). During the experimental setup, we used electrode gel to ensure proper conductivity. EEG signals were sampled at 512 Hz.

### EEG processing

We performed all processing and analysis in Matlab (The Mathworks, Natick, MA) using scripts based on EEGLAB 13.0.1b (http://www.sccn.ucsd.edu/eeglab), an open source environment for processing electrophysiological data (Delorme and Makeig, 2004). We created one EEG dataset for the finger tapping task, and another merged dataset containing EEG data from both walking with eyes open and blindfolded. We high-pass filtered (1 Hz, 8,251 point window, bandwidth 0.2 Hz, passband edge 0.2 Hz) and removed line noise using Cleanline (https://www.nitrc.org/projects/cleanline/) from both datasets. For the walking dataset, we identified the right and left heel strike, and right and left toe-off events for both walking with eyes open and blindfolded conditions from the treadmill ground reaction force using standard methods previously described (Gwin et al., 2011; Kline et al., 2015; Snyder et al., 2015). Briefly, we defined initial foot contacts when the ground reaction force exceeds 15 N and the toe-offs when the force drops below 15 N. Subsequently, we removed channels exhibiting substantial artifacts using the following methods, which combined will be called channel rejection method 1: 1) channels with magnitude < 30 or > 3,000 μV; 2) channels with kurtosis > 5 standard deviations from the mean; (3) channels uncorrelated with the surrounding channels (*r* < 0.4) for more than 1% of the total time; (4) channels with standard deviation qualitatively higher than the other measured channels, verified by visual inspection of the increasingly sorted standard deviation across channels. This method led to a rejection of 23 ± 6.4 channels from the finger tapping datasets, and 37 ± 6.5 channels from the walking datasets using channel rejection method 1.

### Template correlation rejection (TCR)

**Figure 2:**
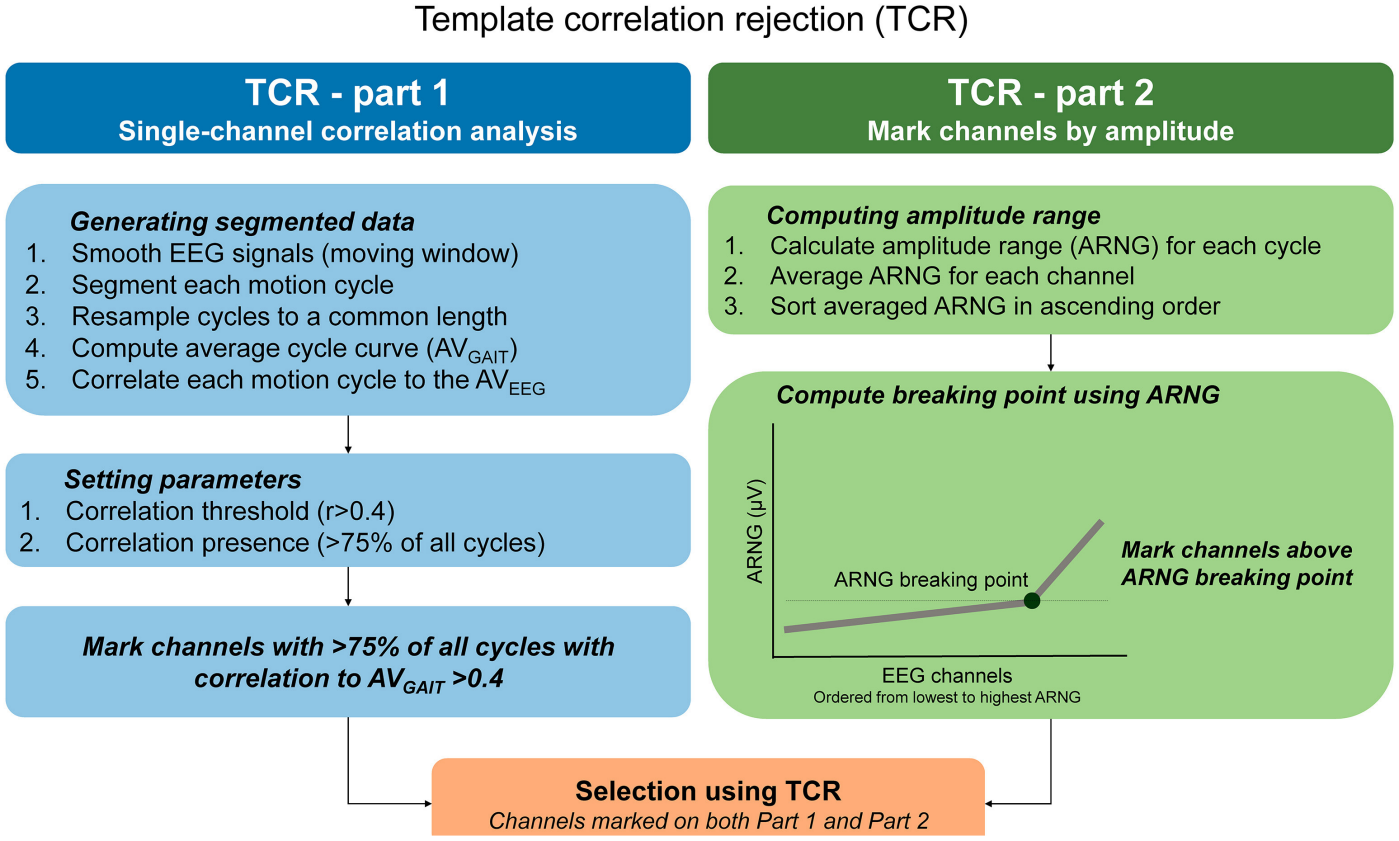
**Step-by-step calculation of the template correlation rejection (TCR) method**. In order to underpin EEG channels carrying motion-related artifacts, the TCR method quantifies the amount of EEG epochs minimally correlated to a channel template, and defines among these channels those exhibiting amplitude ranges (ARNG) unexpectedly higher in comparison to the pool of EEG channels (C).

The TCR method should be applied in EEG datasets free of bad channels selected using standard methods (range, standard deviation, kurtosis, correlation). We illustrated the TCR method for identifying channels influenced by motion artifacts in Figure 2. The basic assumption for TCR is that, for cyclical motions occurring at a steady pace, the head dynamics are repetitive, and EEG motion-related artifacts are repeated throughout the recording. Thus, an EEG channel carrying motion-related artifacts must present a similar amplitude pattern across the majority of individual motion cycles (or epochs). Consequently, these epochs may be correlated to a general EEG amplitude template, created by averaging the EEG data across epochs.

#### TCR part 1—defining correlations

To compute the TCR parameters, we used a copy of the original dataset and applied the following steps for each channel separately: (1) smoothed continuous EEG data using moving average (100 ms windows, 50 ms overlap); (2) data segmentation (from right heel strike to next right heel strike) and time warping of each full gait cycle to a common length. The average gait cycle duration was 1.21 ± 0.4 s, and we resampled all gait cycle epochs to 1,000 points; (3) computation of an averaged template across all time-normalized epochs (AV_GAIT_); (4) computation of Pearson correlation between AV_GAIT_ and each individual EEG epoch representing a gait cycle. Ultimately, this process will return a matrix of correlation coefficients with dimensions equal to the number of gait cycles times the number of EEG channels, for walking with eyes open and blindfolded separately, for each subject.

#### TCR part 2—applying correlation threshold

The next step is to define the number of epochs presenting a relevant correlation to the AV_GAIT_ for each channel. Initially we assumed that, in order to consider a channel as potentially affected by motion artifacts, it should present at least 75% of all epochs (3 out of every 4) minimally correlated to the AV_GAIT_. Preliminary analyses have shown that we could identify the most relevant channels to be rejected by using the threshold set at least at 75% of all cycles.

In order to define relevant correlations, we ran an analysis using the same EEG data during walking with eyes open while substituting the gait events by randomly generated events in time. Our assumption was that motion-related artifacts are locked to some gait events and occur systematically across the EEG recording. Thus, EEG data epoched from random events in time cannot present as many epochs correlated to the AV_GAIT_. For each subject, we generated 10 different EEG datasets, for both the walking with eyes open and blindfolded conditions, containing random epochs for testing the robustness of the analysis. We generated random epochs accounting for 50% of the total number of cycles, in order to reduce the likelihood of overlapping the EEG patterns. We generated a total 520 ± 34 epochs across all subjects. In order to generate epochs fairly across the duration of our recordings, we generated the random timing in 5 different sectors accounting for 20% of the dataset each.

**Figure 3:**
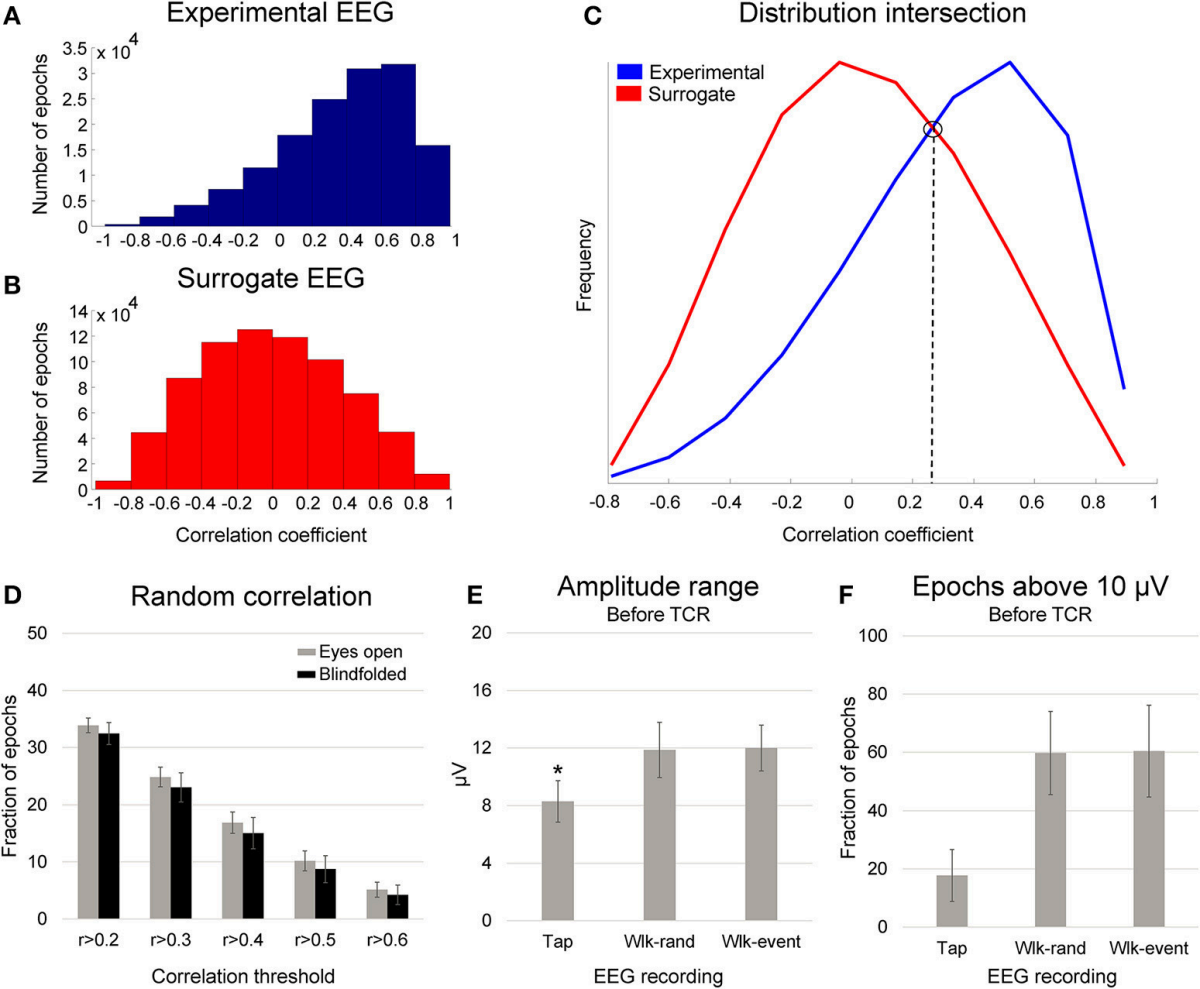
**Distribution of the correlation coefficient between single gait cycle epochs (A**, experimental EEG) and randomly generated epochs **(B**, surrogate EEG) in relation to an average template for one illustrative subject. In **(C)**, the distribution between these two conditions present an intersection point, in which the correlations in the experimental EEG occur above the level of chance. In **(D)**, mean (SD) fraction of total randomly generated epochs (*gait cycles*), from 10 different datasets per subject, above the correlation thresholds from *r* > 0.2 to *r* > 0.6 while walking with eyes open (*gray bars*) and blindfolded (*black bars*). In **(E)**, mean (SD) amplitude range for EEG channels recorded during finger tapping task (Tap) walking with eyes open using randomly generated epochs (Wlk-rand) and using the original gait events (Wlk-event). In **(F)**, mean (SD) fraction of epochs above the threshold of 10 μV the finger tapping amplitude range for the same conditions displayed in the **(B)**. Denotes significant difference in relation to the other conditions (*p* < 0.05).

We performed the same steps previously explained for defining the correlations between random epochs (surrogate dataset) to the AV_GAIT_ for each channel. The distribution of the correlations from the experimental EEG (i.e., gait cycles) was not normally distributed, whereas the surrogate data presents null distribution (Figures 3A,B). By comparing the distribution of the experimental correlations and the surrogate correlations, it is possible to define an intersection point (see right figure above). This point describes when the experimental correlation distribution still rises, whereas the surrogate data distribution is falling (Figure 3C). Therefore, this intersection point indicates the level in which the experimental correlations are happening above the level of chance from the null distribution. The Pearson correlation values at the intersection point were *r* = 0.33 ± 0.09, and we determined a correlation threshold by rounding up this average number to *r* > 0.4.

In order to evaluate whether this proposed correlation threshold would be appropriate, we computed the fraction of epochs distributed in correlations ranging from 0.2 to 0.6 (illustrated in Figure 3D). The proposed correlation analysis using random epochs showed that the fraction of epochs reaching correlations above 0.2 is ~33%, and the fraction is reduced as a function of higher correlation thresholds. In addition, we found that by using random epochs there are only <20% of the total epochs with correlation coefficient *r* > 0.4. Therefore, EEG channels presenting >75% of all epochs with correlation *r* > 0.4 to the AV_GAIT_ cannot be caused by chance; rather, it is a consistent temporal pattern across the recording that can be evidenced by segmenting epochs using motion events.

#### TCR part 3—defining relevant EEG amplitude

The degree of correlation to a template is essential for defining the influence of cyclical motion in specific channels. However, the modulation of motor actions may be occurring at strictly repetitive instants throughout the motion cycle. This means that the exclusion of channels based only on the template correlation may exclude meaningful, artifact-free channels. Therefore, it is vital to further evaluate the content of such channels. We hypothesized that differences in EEG amplitude between highly controlled recordings in the seated and walking conditions could contribute to define an amplitude threshold in addition to the correlation threshold, as higher amplitudes during walking could be caused by artifacts rather than brain signals.

We compared the EEG amplitude of channels recorded during walking in comparison to the finger tapping task. The assumption was that the amplitude from channels recorded during finger tapping would be substantially lower in comparison to the noisy channels recorded during walking. We created epochs of 1 s around the finger tapping for calculating the amplitude ranges. For both the finger tapping and walking with eyes open and blindfolded tasks, we computed the amplitude range across windows of 10% epochs, averaged the values to represent the epochs for each channel, and subsequently averaged all the channels for representing each subject. By subdividing the gait epoch in smaller windows we reduce the influence of large fluctuations caused by short spikes on the EEG data throughout the total number of epochs. In this way, epochs presenting high amplitude range are those containing large fluctuations throughout the epoch length. We used a 1-way ANOVA for assessing the differences in amplitude range between finger tapping and walking using random events x walking using motion events (Figure 3E). Additionally, we used a Tukey HSD *post-hoc* test for defining differences between pairs of conditions, and the significance level was set at *p* < 0.05.

We observed ~33% increase in EEG amplitude range between both walking conditions in relation to the finger tapping task (*p* < 0.001). No differences were found between the walking conditions. The upper bound of the 95% confidence interval for the finger tapping amplitude range was ~10 μV. We found that ~17% of the channels from the finger tapping EEG showed average amplitude range higher than 10 μV (Figure 3F). On the other hand, ~59% of the channels from EEG during walking showed amplitude range higher than 10 μV, regardless of the events used for epoching. This result indicates that EEG channels exhibiting amplitude range ~10 μV or lower cannot be immediately associated with motion artifact, since EEG data recorded in seated conditions can reach similar amplitudes. Therefore, it is not suitable to use a fixed threshold for defining EEG channels affected by motion-related artifacts.

#### Defining amplitude breaking point for EEG channels

Instead of using a fixed amplitude threshold, we opted for computing a “breaking point” on the EEG amplitude range across channels. We used a Matlab function freely available online to define the knee point distribution: (http://www.mathworks.com/matlabcentral/fileexchange/35094-knee-point/content/knee_pt.m).

**Figure 4:**
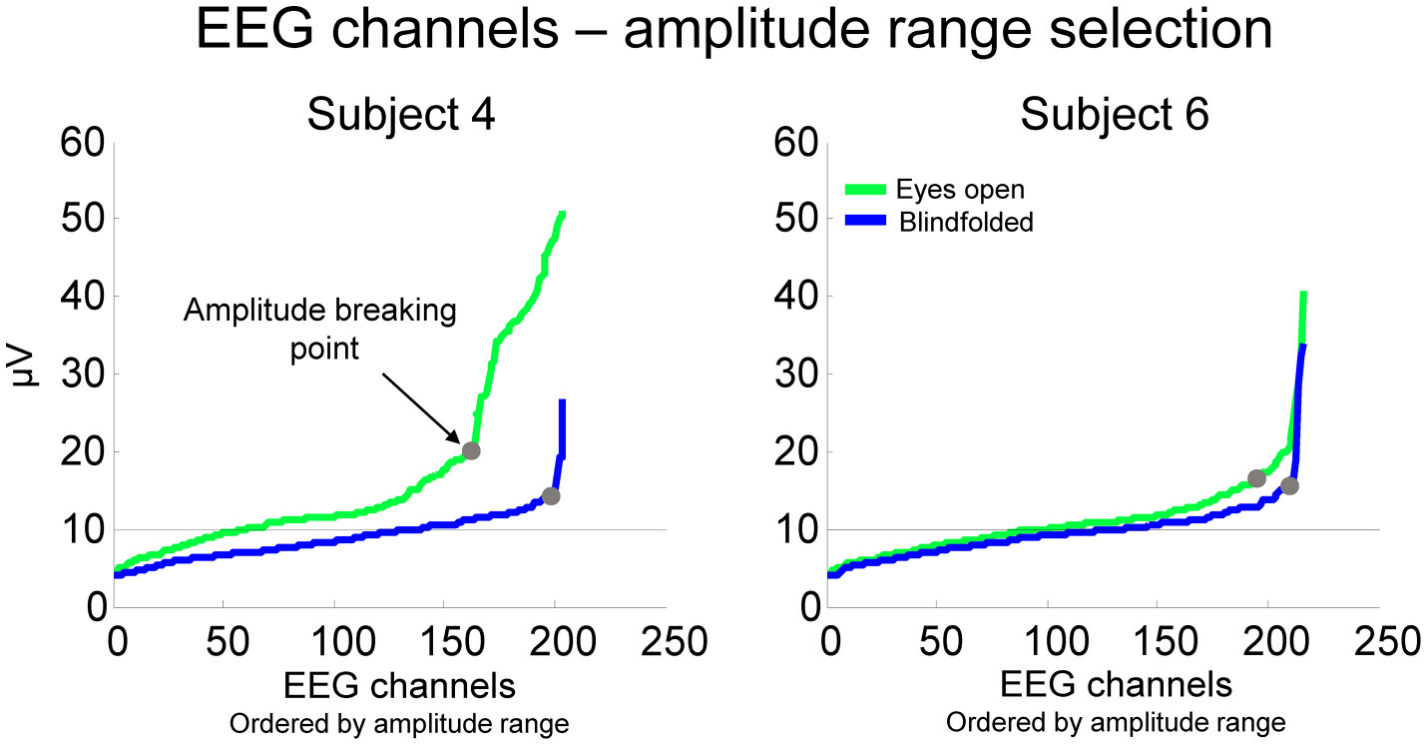
**EEG amplitude range for all channels ordered from the lowest to the highest amplitude for two representative subjects while walking with the eyes open (green lines) and blindfolded (blue lines)**. We established that channels potentially considerable for rejection were those located at the right side of the point at which the EEG amplitude of channels start to increase substantially (breaking point).

This method has the advantage of being specific to the distribution of EEG amplitudes from each recording. For the calculation, we ordered the EEG channels from the lowest to the highest amplitude range and computed the point in which the amplitude range starts to increase steadily (Figure 4). The breaking points for all subjects were at 16.3 ± 5.1 μV for walking with eyes open and 12.6 ± 2.5 μV for walking blindfolded, which were higher than the average amplitude range found for finger tapping (~10 μV in this study).

Ultimately, the TCR considered channels containing motion-related artifacts as those presenting >75% of all EEG epochs correlated at *r* > 0.4 and located above the amplitude breaking point from both walking with eyes open and blindfolded conditions. In case of channels being selected only for the walking EO condition, we also removed this channel from the walking blindfolded condition and vice-versa. This concludes the second step of the TCR method. In the following sections, we demonstrate features of the TCR method in comparison to standard rejection methods and the efficacy of TCR in identifying motion-related independent components.

## Results

In this section, we describe statistical analysis and show results on the differences between rejected channels and clean channels in section Absolute Power of Removed Channels, as well as the effectiveness of TCR in identifying independent components carrying motion-related artifacts.

### Profile of EEG channels marked using TCR

**Figure 5:**
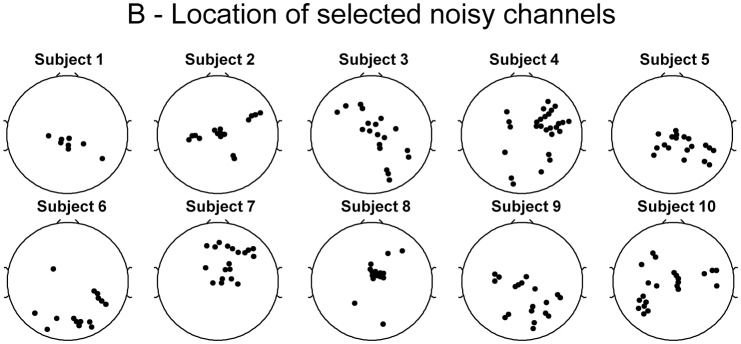
**Spatial representation for each subject of the location of channels marked for rejection from EEG datasets using the template correlation rejection method**.

The TCR method marked 22 ± 9 channels for rejection; these showed no robust location pattern across subjects (Figure 5). Most subjects had marked channels at the center of the head, whereas the locations on the sides, front and back of the head were subject-specific. This is a first indication that TCR labeled channels do not represent neuronal activity but might be representing motion-related artifacts.

### Absolute power of removed channels

We performed all further data analysis using the original EEG datasets prior to computing TCR, therefore all processing related to the TCR method (moving window, time-warping) does not influence our results. We used the channels selected from TCR to generate EEG datasets from walking with eyes open and blindfolded in order to investigate the properties of the rejected channels. We computed the absolute power using 512 ms window size, 1,024 ms zero-pad from channels or independent components in the delta (1–4 Hz), theta (5–8 Hz), alpha (9–13 Hz), beta (14–30 Hz), and gamma bands (31–80 Hz). For each subject, we determined the median absolute power across all channels/independent components in each frequency band. In order to perform statistical analysis, we used 1-way ANOVA and subsequent Tukey HSD *post-hoc* test for assessing the differences in absolute power between different stages of the channel rejections (channel rejection method 1 vs. TCR vs. the remaining good channels after using both these methods) in each frequency band. The significance level was set at *p* < 0.05.

**Figure 6:**
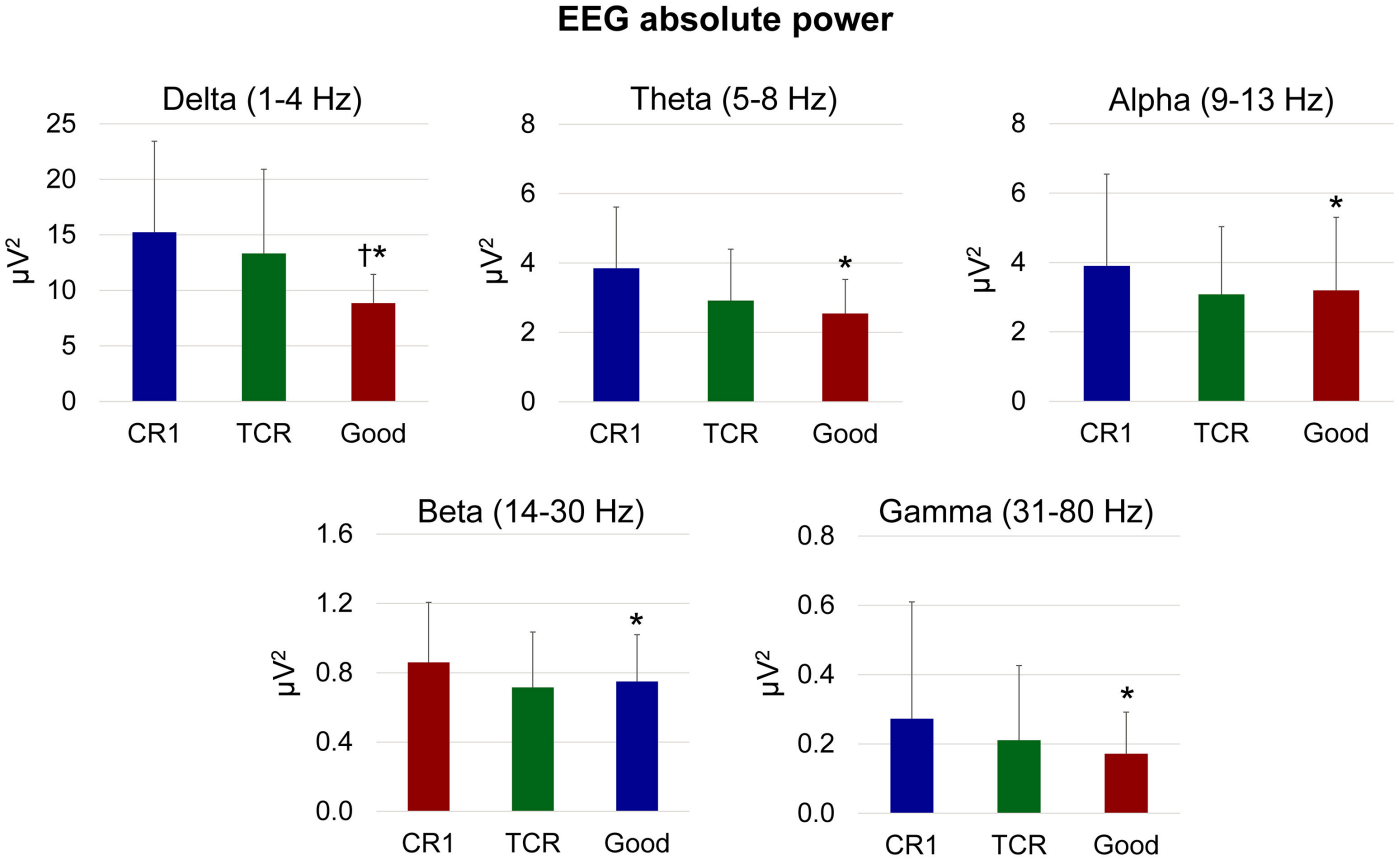
**Mean (SD) absolute power in different EEG frequency bands from channels rejected using traditional methods (CR1), using the proposed template correlation rejection (TCR) method and the remaining good channels after rejecting all other marked channels (Good)**. ^*^Denotes significant difference in relation to CR1 (*p* < 0.05); ^†^Denotes significant difference in relation to TCR (*p* < 0.05).

There was a significant effect of frequency band on the absolute power computed from the EEG channels in the different stages of channel rejection [*p* < 0.001, *F*_(4, 135)_]. More importantly, there was a significant effect of the different stages of the channel rejections (channel rejection method 1 vs. TCR vs. good channels) on the absolute power [*p* < 0.05, *F*_(2, 135)_, Figure 6]. The absolute power from the channels rejected using channel rejection method 1 was higher than the absolute power from the good channels for all frequency bands. In addition, the power from TCR at the delta band was ~60% higher in comparison to the power of good channels (*p* < 0.01). There were no further differences between TCR and good channels.

### Independent components from channels rejected using TCR

**Figure 7:**
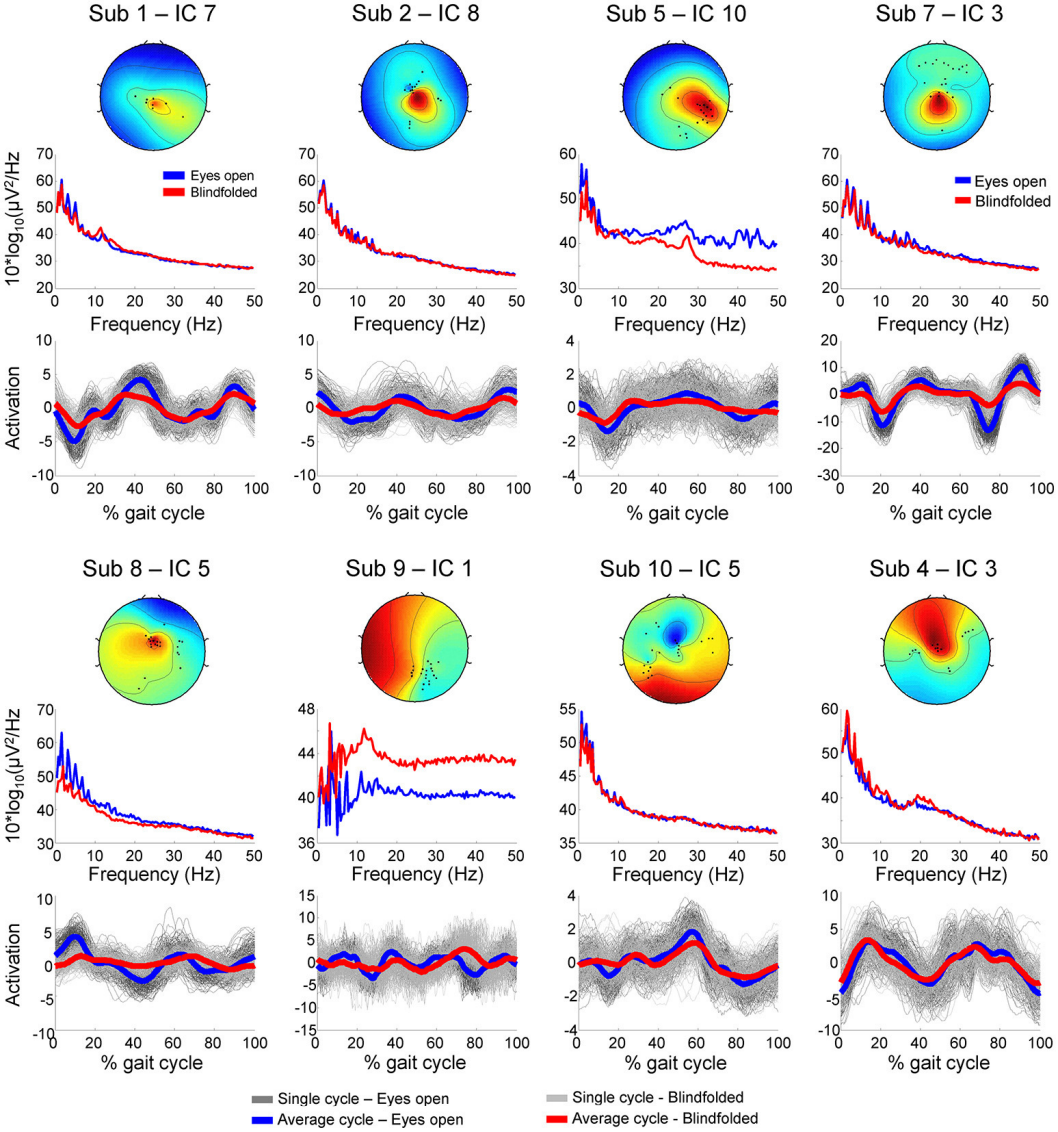
**Scalp maps, power spectrum and independent component activation signals from eight representative independent components extracted from the channels marked for rejection using the template correlation rejection method (TCR) for both eyes open (blue) and blindfolded condition (red)**.

**Figure 8:**
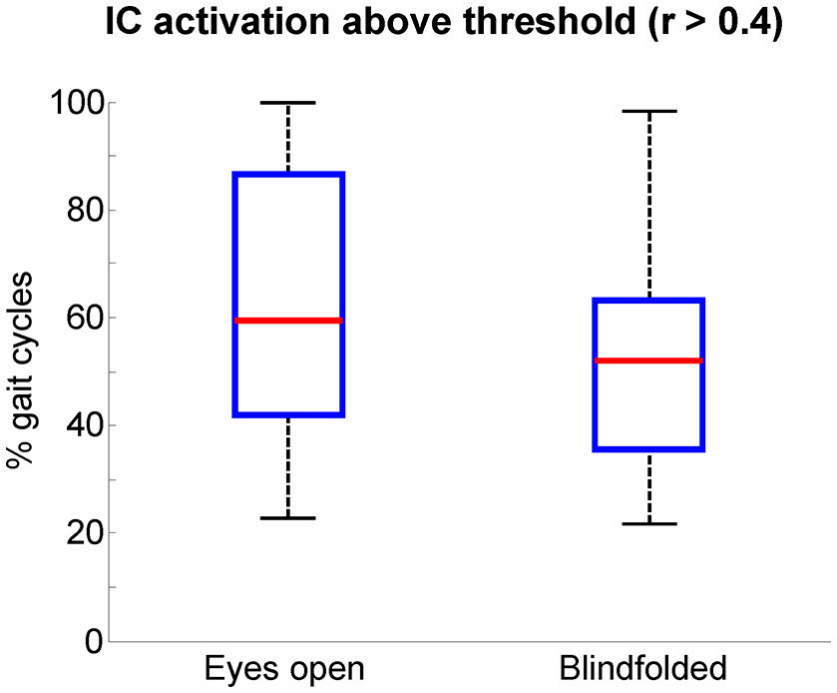
**Boxplots of the percentage of gait cycles with correlation ***r*** > 0.4 with respect to the average template gait cycle for the activation signals of independent components extracted from the channels marked for rejection using the template correlation rejection method (TCR)**. The central red mark is the median, the edges of the box the 25th and 75th percentiles. The whiskers cover approx. 99% of the data.

We assumed that EEG channels carrying motion-related artifacts contribute to the generation of artifactual independent components. The spectral properties of these independent components carrying time-locked motion artifacts can be interpreted as neural modulation or contribute to build event-related spectral perturbation plots representing misleading results. In order to provide evidences on the influences of channels selected using TCR when reporting EEG results using event-related spectral perturbation analysis, we isolated these noisy channels as EEG datasets (merging walking with eyes open and blindfolded conditions) and performed traditional EEG processing using ICA. The rejected channels generated 60 independent components with residual variance below 15% located in brain. The power spectrum of these independent components usually presented jagged pattern for frequencies below 20 Hz for both eyes open and blindfolded conditions (Figure 7). In addition, the independent component activation signals showed temporal patterns coupled to the gait cycle. We found a median value of 60% of all gait cycles with correlation above the threshold *r* > 0.4 for the eyes open condition (Figure 8), whereas for the blindfolded condition this percentage was reduced to 50%.

### Cluster analysis from channels rejected using TCR

We used the independent components extracted from channels rejected using TCR to generate clusters, and subsequently represent the data using event-related spectral perturbation plots. We clustered the independent components from all subjects using a k-means clustering algorithm available in EEGLAB for the walking with eyes open and blindfolded separately. We prioritized the clustering by similar power spectrum and scalp maps and computed event-related spectral perturbations based on the clustering. The methods for time-warping EEG epochs, subtracting baseline for each condition and computing significant differences in relation to baseline frequency are described in previous work from our research group (Gwin et al., 2011; Kline et al., 2015). Note that for the ERSP spectrum visualizations, data were significance masked, meaning all non-significant regions were set to zero.

**Figure 9:**
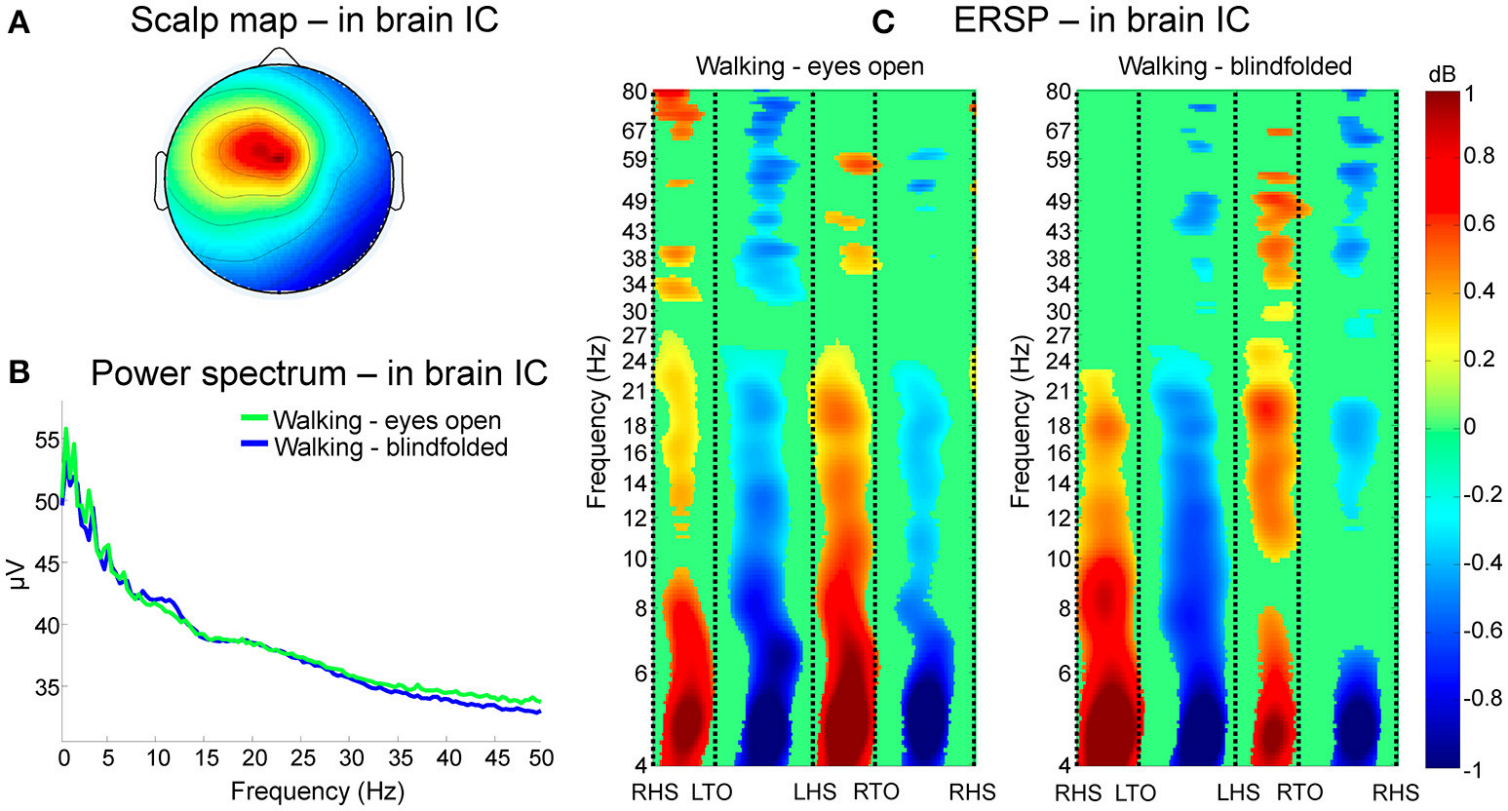
**Clustering of channels marked for rejection using the TCR method**. In **(A)**, scalp maps of a representative cluster of in-brain independent components (ICs) presenting dipolar activity at the center of the head across eight subjects. The power spectrum of these clustered independent components **(B)** show jagged pattern at frequencies below 10 Hz for both eyes open (*green line*) and blindfolded conditions (*blue line*). Analysis of event-related spectral perturbation (ERSP, **C)** show broadband spectral modulation of the contained artifacts. There is a consistent and significant event related synchronization and desynchronization locked to the gait phases from theta to beta bands for both walking conditions.

An illustrative cluster of independent components generated from channels rejected using TCR is shown in Figure 9, where 16 components from eight subjects are represented. This cluster has dipolar properties approximately at the center of the scalp map (Figure 9A). The power spectrum of the clustered independent components presented jagged pattern for frequencies below 15 Hz, and overall similar curve patterns between walking with eyes open and blindfolded (Figure 9B). The use of ERSP plots revealed strong event-related synchronizations immediately after right and left heel strikes at delta, theta, alpha and beta bands, and event-related desynchronization during single-support phases of walking (Figure 9C). Moreover, we found reduced power changes for the blindfolded condition especially in the second half of the gait cycle. These results combined suggest that TCR may be effective in identifying channels carrying motion-related artifacts that are common to the majority of subjects and that can introduce undesired power in frequencies below 25 Hz.

### TCR for removing independent components carrying motion-related artifacts

After rejecting channels marked using TCR from the merged walking with eyes open and blindfolded EEG datasets, we applied infomax ICA on these datasets in order to parse EEG signals into maximally IC processes. The use of TCR for rejecting channels may not remove all motion-related artifacts, and the use of this method for identifying components carrying motion-related artifacts can reduce the influence of these artifacts on the interpretation of EEG analysis. Our assumption was that TCR could identify independent components carrying motion-related amplitude patterns just as performed at the channel level. Subsequently, these independent components may be classified as artifacts if their characteristics of power spectrum and scalp maps do not suggest neural content.

The method consists of applying TCR parts 1 and 2—explained in sections TCR Part 1—Defining Correlations and TCR Part 2—Applying Correlation Threshold—on the independent component activation vectors instead of EEG channels. For rejecting independent components, we disregarded the TCR part 3 explained in the section TCR Part 3—Defining Relevant EEG Amplitude, which is related to computing changes in EEG amplitude. We skipped this step because the unit for independent components are normalized and may not be comparable across components. After identifying potential independent components for rejection, we evaluated their spectral power and scalp maps following recent recommendations reported by Chaumon et al. (2015) for identifying features of neural independent components. These features were: (1) power peak(s) at physiologically relevant frequency bands such as alpha, beta or gamma bands and (2) scalp maps presenting smooth/dipolar topography. In this way, we aimed at defining if the selected components were neural rather than trying to evaluate if they were not neural. In the case of components being evaluated as neural, we did not reject this component from the EEG dataset. On the other hand, all other independent components were marked for rejection as they contained gait-related amplitude patterns and there was no evidence that they carried neural content.

**Figure 10:**
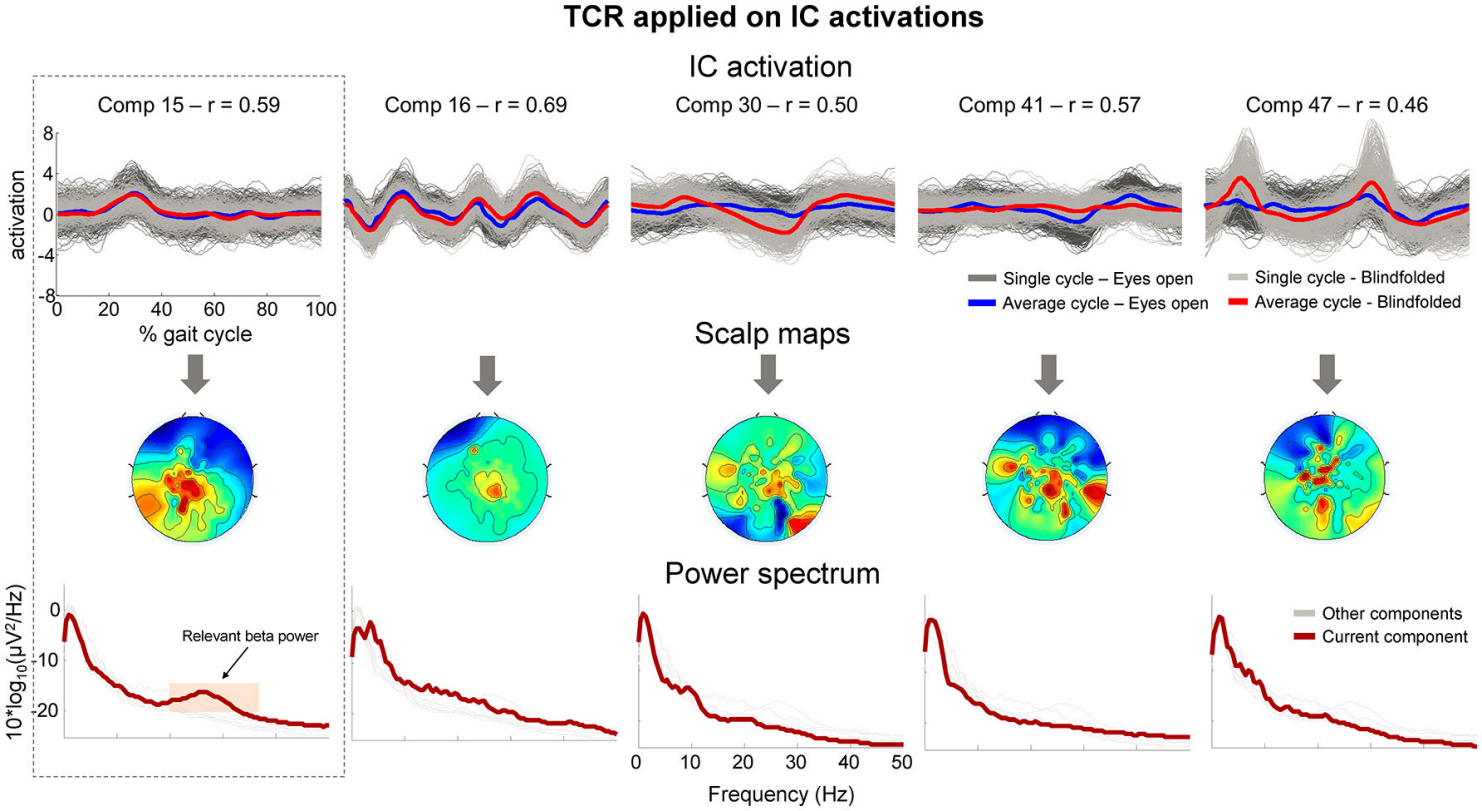
**Properties of independent components (ICs) marked for rejection using the proposed TCR method**. The upper row shows the time-normalized amplitude pattern of the independent components while walking with the eyes open (*dark gray and blue lines*), and walking blindfolded (*light gray and read lines*). The scalp maps (*middle row*) do not show smooth dipolar topography and the independent components power spectrum (*bottom row*) do not show clear power peak at physiologically relevant frequencies. There is an exception for Comp 15 (first column), which show relevant power peak at approximately 25 Hz (*orange shaded area)*. The presence of the relevant power peak indicated that the component should not be rejected from the EEG dataset.

After applying the TCR method for rejecting channels (part 1) and components presenting gait-related motion artifacts (part 2), we found 4.3 ± 1.8 independent components per subject for potential rejection. In Figure 10 (*upper row*), we show the segmented IC activation traces of a sample subject from the independent components classified as containing motion-related amplitude fluctuation (*r* > 0.4). The scalp maps for all these components (Figure 10, *middle row*) did not exhibit the dipolar pattern expected for neural independent components. On the other hand, the power spectrum curve (Figure 10, *bottom row*) for component 15 showed relevant power increase at the beta band (20–35 Hz, shaded area), suggesting that this component carried neural information, and therefore we declined to reject this component from the EEG dataset. All other components did not show relevant spectral properties and presented the jagged pattern below 10 Hz, which was also found in the cluster of bad channels in Figure 9B.

**Figure 11:**
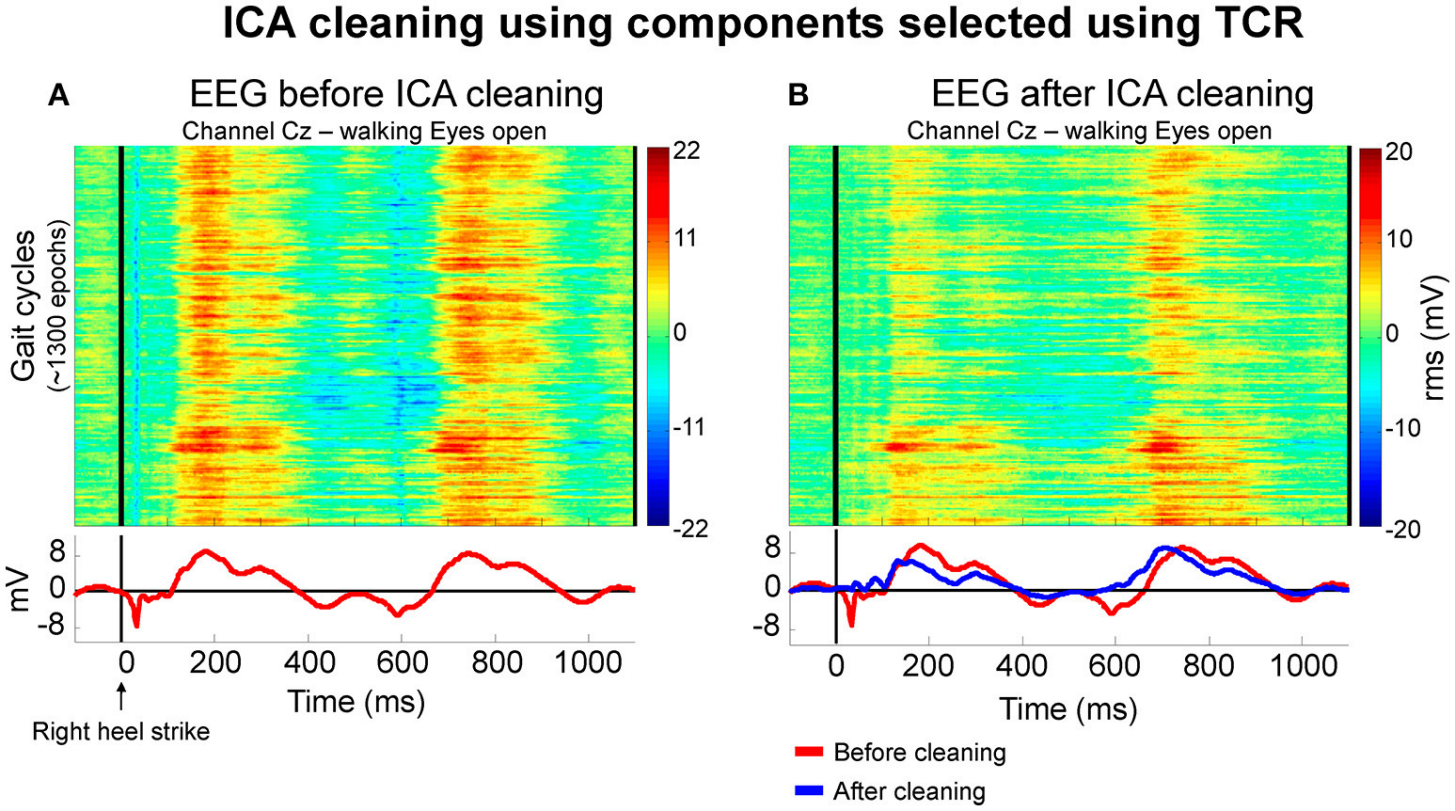
**Color maps illustrating the EEG amplitude (in RMS μV) from approximately 1,300 gait cycles of a representative subject before (A)** and after independent component analysis (ICA) cleaning using independent components selected using the proposed template correlation rejection (TCR) method. Before cleaning, the averaged EEG amplitude (*bottom row, red lines*) presented localized EEG reduction immediately after right heel strike. After rejecting components, the average amplitude pattern **(B** *at the bottom, blue line*) presented reduction in amplitude fluctuation related to initial foot contact to the ground.

After rejecting bad components, we generated ERP images shown in Figure 11. These ERP images contain the segmented walking EEG data from the channel Cz before (Figure 11A) and after cleaning (Figure 11B) by removing the independent components identified using the TCR method for an illustrative subject. A qualitative comparison between the plots suggests slightly reduced overall EEG amplitude after cleaning. In addition, there were abrupt EEG amplitude reductions at approximately 40 and 600 ms before EEG cleaning by rejecting independent components. Both instances represent the immediate changes related to right and left initial foot contact to the treadmill belts, respectively. After rejecting independent components containing motion-related artifacts, the same channel Cz showed slightly lower amplitude in the color map on the right. Moreover, the rejection of the selected independent components was able to attenuate/remove the abrupt changes in EEG amplitude immediately after initial foot contact to the treadmill belts. Despite the success in attenuating motion-related artifact content, other source of artifacts such as electromyographic activity and electro-ocular activity may still be present in our datasets (Gwin et al., 2010).

**Figure 12:**
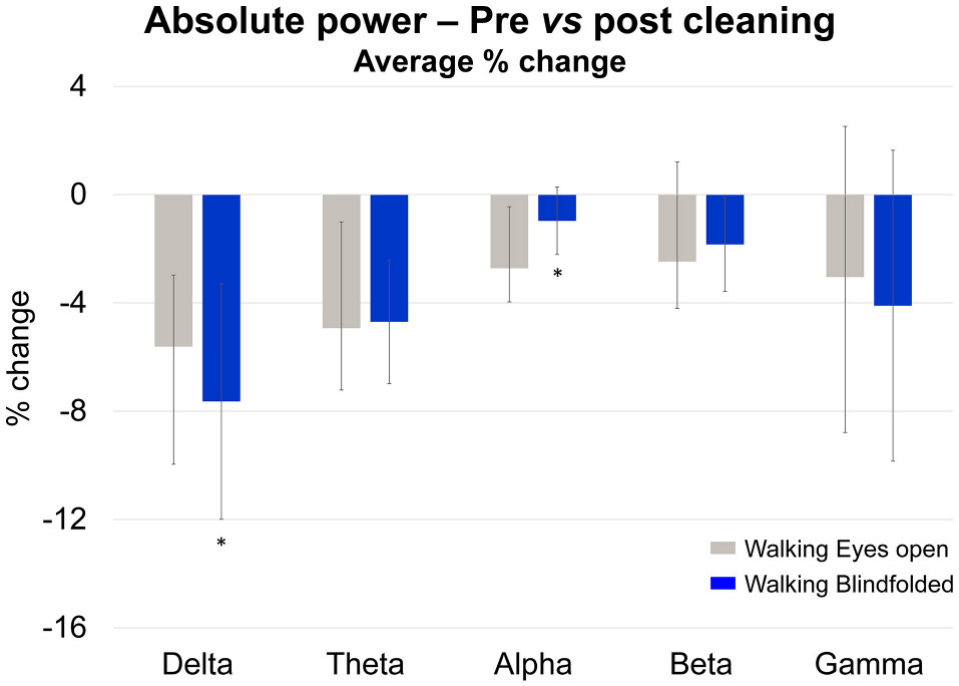
**Mean (SD) percentage of change (% change) in absolute power calculated at the delta, theta, alpha, beta, and gamma bands while subjects walked with the eyes open (***gray bars***) and blindfolded (***blue bars***)**. ^*^Denotes significant differences in relation to walking with eyes open (*p* < 0.05).

In order to describe the effect of rejecting independent components identified using the TCR method in all remaining channels, we analyzed the changes in absolute power from before- to after-cleaning (Figure 12) using the same methods described in section Absolute Power of Removed Channels. We used a 2-way ANOVA for assessing the effects of walking condition (eyes open vs. blindfolded) and cleaning (before vs. after cleaning). The significance level was set at *p* < 0.05. For presenting the results, we normalized the power as a percentage of change in relation to the power before rejecting independent components.

The delta and theta bands showed 4–8% reductions in absolute power after cleaning. However there was no statistical effect of cleaning on the absolute power for all frequency bands (*p* > 0.05). On the other hand, there was a significant reduction in the delta absolute power for the blindfolded condition when compared to the eyes open condition [*p* < 0.05, *F*_(1, 36)_], as well as significant reduction in the alpha absolute power for the walking with eyes open condition when compared to the blindfolded condition.

### Comparing neural ICs extracted with and without TCR

**Figure 13:**
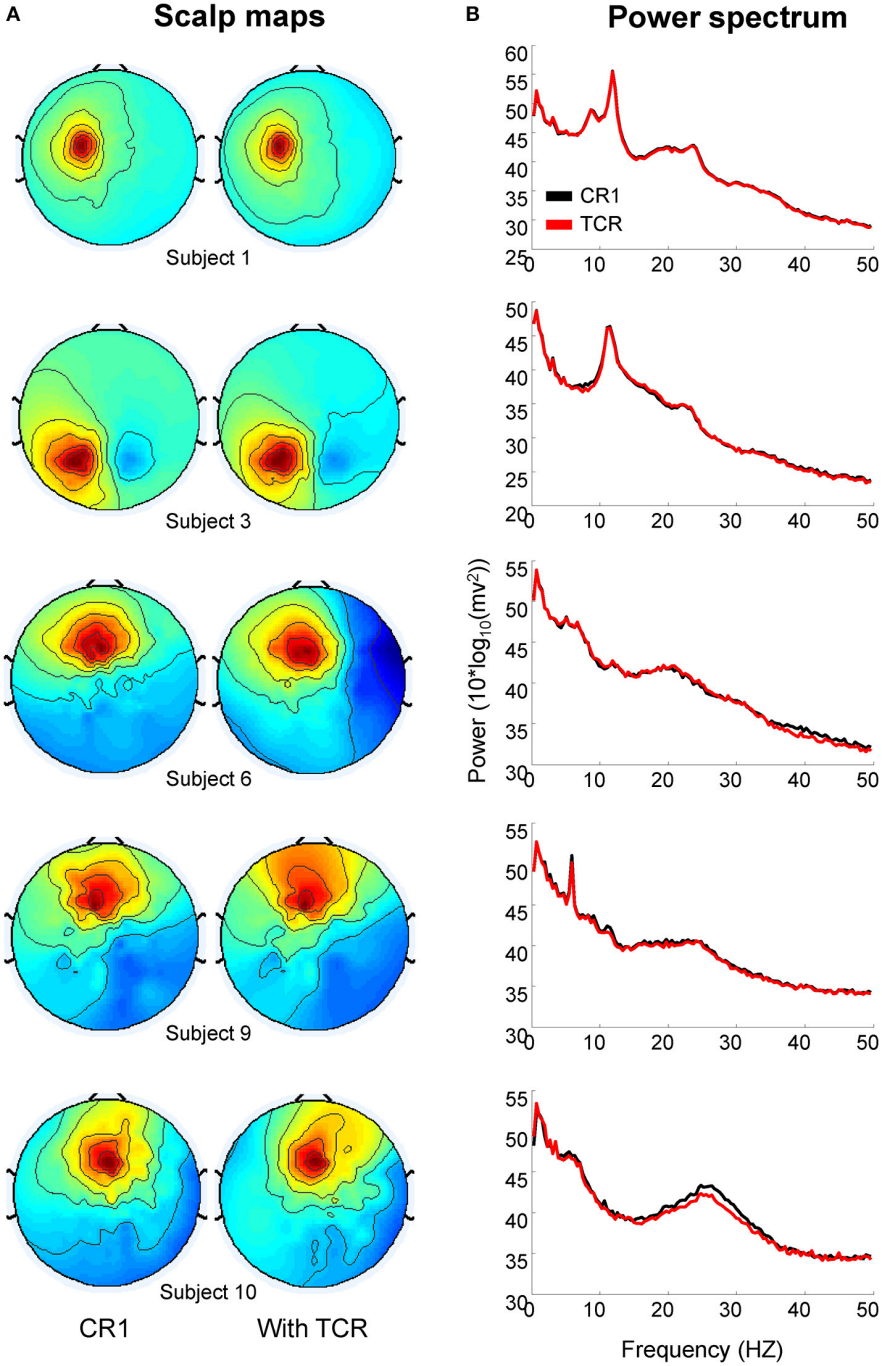
**Scalp maps (A)** and power spectrum **(B)** of independent components extracted from EEG datasets processed without the template correlation rejection (TCR) and with the TCR method.

In order to validate the method, it is important to assure that the neural content of the EEG dataset is not compromised. We have selected two homologous independent components from the dataset analyzed without TCR (e.g., only rejecting channels by using the traditional channel rejection methods) and the dataset with TCR processing for each subject. Subsequently, we compared the changes in power spectrum related to the processing method. Figure 13 shows illustration of five pairs of independent components, in which it is possible to observe nearly identical scalp maps and power spectrum curves between the two processing methods.

**Figure 14:**
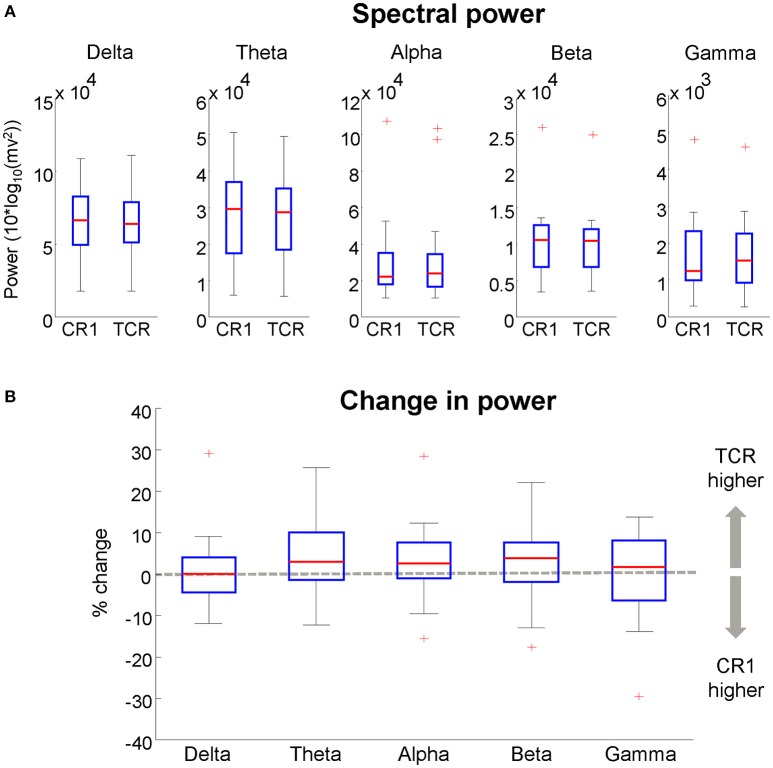
**(A)**, boxplots of the spectral power of neural independent components extracted from datasets processed using traditional channel rejection (CR1) and using the TCR method to remove channels and independent components containing motion-related artifacts (TCR). In **(B)**, relative difference in spectral power between CR1 and TCR for different frequency bands. For each box, the central red mark is the median, the edges of the box the 25th and 75th percentiles. The whiskers cover approx. 99% of the data. ^+^Denotes samples outside the boxplot limits.

We computed the absolute power from the independent components using the same methods described in section Absolute Power of Removed Channels. There was no difference between the absolute power of components extracted without applying TCR and when applying TCR for all frequency bands (Student *t*-test, Figure 14A *p* > 0.05). Moreover, for all frequency bands, the median percent change between the two methods was below 6% across all investigated independent components (Figure 14B). These results suggest that the TCR method does not compromise the identification of relevant/neural independent components, both in terms of scalp maps and spectral power.

## Discussion

Our results indicated that the template correlation rejection method was able to identify EEG channels and independent components that had substantive motion-related artifacts. Channels marked for rejection this method presented higher absolute power at the delta band in comparison to good channels. In addition, independent components generated from these noisy channels presented oscillations in the spectral power below 20 Hz and activation coupled to the gait cycle. These components formed a cluster presenting consistent power changes time-locked to the gait events at frequencies below 25 Hz, both while subjects walked normally or blindfolded. Maintaining these noisy EEG patterns through the final analysis could introduce undesired time-locked changes in spectral properties of event related spectral perturbations when combined with good channels. The proposed cleaning method would slightly reduce the EEG power spectrum (1–8%) in relation to EEG data processed using only traditional channel rejection methods. The effect was more pronounced in conditions with greater head displacement when walking normally in comparison to walking blindfolded. Nonetheless, the content of components classified as carrying relevant neural information would not be compromised, as we found no significant changes in the absolute power of neural components extracted with and without using the template correlation rejection method. While it is possible that rejecting the noisy independent components from the dataset could eliminate true electrocortical sources from the analysis, it would be the conservative approach to attenuate motion artifacts in EEG studies. In data from our study, we found the main influence of motion artifact during human locomotion came during the abrupt amplitude changes related to initial heel strike. When analyzing data from other repetitive human movements, such as cycling, rowing, or hopping, our method would also serve to reduce the influence of motion dynamics on scalp EEG artifacts.

Motion-related artifacts in EEG recordings during whole-body motion may be primarily identified by a higher amplitude range of noisy channels. Gwin et al. (2010) has shown that slower walking speed reduce the occurrence of artifacts on low EEG frequencies (i.e., theta band). However, there is a lack of normative data with respect to differences in EEG amplitude between clean stationary EEG data and EEG acquired during walking. In this study, we addressed this issue by recording EEG during a rhythmic finger tapping task that evoked motor potentials and comparing it to the EEG recordings during walking. After rejecting channels using channel rejection method 1 in both conditions, we found that EEG amplitudes during walking are ~33% higher than those from stationary recordings. Approximately 17% of all EEG channels from finger tapping were above a pre-established threshold of 10 μV, whereas ~40% of all EEG channels during walking were below this threshold. Therefore, defining a fixed threshold based on the stationary recording may not be ideal because it may erroneously mark good channels for rejection. Notably, using a fixed-threshold criterion can be problematic because replication of results can be influenced by many factors related to motion (walking/running speed) as well as differences in the EEG equipment used for recordings (for instance, differences in reference sites or electrode impedance can affect overall amplitude values). Nonetheless, we were able to use a repeatable, objective measurement technique to successfully determine a breaking point for EEG amplitude across channels for both walking with eyes open and blindfolded at the same speed, which can be applied in different motion patterns. The determination of the breaking point allows to apply this method in different EEG setups (e.g., different EEG equipment, number of channels, repeated experiments), while still maintaining an objective criterion for defining the amplitude threshold.

Attempts at quantifying and reducing motion artifacts in scalp EEG during cyclical motion has been addressed in different ways. Gwin and co-workers in two studies (Gwin et al., 2010, 2011) have used a template subtraction method that may reduce the influence of motion artifacts from independent components. Onikura et al. (2015) recently proposed a cleaning method based on the correlation of head accelerometry and individual independent components. Both these methods achieve some success, but they also have limitations. The template subtraction can be deleterious since the template may remove relevant neural information from the EEG dataset. In the case of Onikura et al. (2015), the limitation is that head accelerometry is not correlated uniformly to all EEG channels (Kline et al., 2015), because the six degree of freedom motion of the head influences individual EEG channels differently (Kline et al., 2015; Costa et al., 2016). These limitations emphasize the difficulty in defining a single ideal method for reducing motion artifacts during EEG recordings. Regarding spectral analysis, Seeber and co-workers (Seeber et al., 2015) have created a frequency clustering method to separate muscle activity artifacts (especially from neck muscles) from neural activity. Muscle activity is usually broadband, and previous studies have shown motion artifacts being present in low frequencies (Castermans et al., 2014). Our study provides evidence that motion-related artifacts may occur from 4 H to 24 Hz, also occurring even in higher frequencies between 30 and 80 Hz (Figure 9). Further studies can evaluate whether frequency clustering method is also feasible to identify motion-related artifacts during walking.

Nathan and Contreras-Vidal (2016) investigated motion artifacts during walking by applying artifact subspace reconstruction for removing artifacts. The authors also performed wavelet coherence analysis at the delta band (0.1–4 Hz) between one accelerometer and three different EEG channels for showing the reduced artifact content on these channels. The authors concluded that motion artifacts during locomotion are negligible for walking speed below 4.5 km/h (1.25 m/s). It is noteworthy that using a single six degree-of-freedom accelerometer for validating the success of a cleaning method may be questionable. EEG recordings throughout different scalp regions present different acceleration patterns as described in Kline et al. (2015) and illustrated in our Figure 1B. Moreover, the focus of the coherence analysis only on the delta band must be taken into account, as there is no data supporting the absence of motion artifacts in other higher frequency bands. Our results have shown that independent components carrying motion-related artifacts can influence spectral power up to 25 Hz (theta, alpha, and beta bands), and at a lesser extent the gamma band (>30 Hz). Other research groups have suggested that motion artifacts may be present at frequencies up to 60 Hz (Castermans et al., 2014).

It is important to emphasize that we used an active electrode EEG system (Biosemi Active Two) and took all precautions cited by Nathan and co-workers in order to minimize cable movements and reduce motion artifacts on the EEG recordings. Nonetheless, we still found a considerable amount of channels being influenced by motion artifacts. In addition, the use of artifact subspace reconstruction requires a clean baseline for identifying noisy sectors of data. Nathan and co-workers do not mention which type of data were used as baseline, and reported data of three EEG channels from only three subjects. The results from Nathan and Contreras-Vidal (2016), may point to a possible direction for EEG cleaning, but more research is needed to reinforce the results. Previous studies have shown that motion artifact is a major to investigate EEG during locomotion (Castermans et al., 2014; Reis et al., 2014; Kline et al., 2015; Snyder et al., 2015; Oliveira et al., 2016a), and the present results demonstrated that not rejecting channels carrying motion-related artifacts can influence the results and interpretations of EEG processing.

There were no differences between neural independent components extracted with and without using the TCR method. Therefore, the proposed method may not compromise the identification of relevant/neural independent components, both in terms of scalp maps and spectral power. This result raises a question: What is the relevance of the proposed cleaning method if it is possible to identify neural components without applying such method? The TCR method has the potential to remove channels that can generate misleading independent components, or add undesired power into neural components. The channels identified using the TCR method can generate independent components containing gait-related motion artifacts (see Figure 7), and removing these channels may be crucial to assure appropriate interpretation of electrocortical activity during cyclical whole-body movements. Therefore, the advantage of using TCR is the possibility of minimizing the potential inclusion of misleading components into the EEG data processing and further interpretations.

Analyzing uncorrupted channels is undoubtedly crucial to minimize the influences of motion artifacts on the interpretation of EEG results. We believe that the first action toward this direction is the identification of channels influenced by motion, not allowing such channels to be present during independent components analysis processing. We used a high-density EEG system with 256 active electrode channels. The rejection of 15–20 channels influenced by motion artifacts may not compromise the quality of the final EEG dataset or the independent components extracted from such a high number of remaining channels. The use of a large number of channels also optimizes the identification of amplitude breaking points, which is essential for successful identification of channels containing high amplitudes. On the other hand, the use of EEG systems equipped with low numbers of channels (64 or below) may impose a limitation to optimal use of the TCR, as the rejection of 2–3 neighboring channels can substantially reduce the acquired information from target brain areas. Recent investigation has shown that the signal-to-noise ratio calculated from independent components increases linearly as a function of the number of recorded EEG channels (Oliveira et al., 2016a). Therefore, we recommend the use of as many EEG channels as possible for maximizing the acquisition of relevant neural data.

The use of TCR for rejecting independent components initially identifies any independent components containing time-locked amplitude patterns related to the motion event, and the rejection of such independent components without further evaluation can erroneously remove independent components containing relevant neural information. Recently, Chaumon et al. (2015) have published a comprehensive guideline for identifying relevant neural independent components and artifactual independent components from EEG data recorded in stationary conditions. After identifying motion-related independent components in our datasets, we referred to their description of neural independent components to identify which independent components should be maintained in the EEG datasets. To date, there are no guidelines on how to identify independent components carrying motion-related artifacts. The present study exemplifies the power spectrum and scalp maps of independent components carrying motion-related artifacts and also the event-related spectral perturbation properties of a cluster of these independent components across multiple subjects. More investigations describing problematic independent components in different types of cyclic motion (walking, running, cycling) in different speed may help establishing the expected pattern of problematic components.

In the present study we described a novel method for rejecting channels and components carrying motion-related artifacts from two walking tasks performed at an identical speed (1.0 m/s). In addition to normal walking, subjects also walked blindfolded for inducing changes in walking pattern that could attenuate the influence of motion artifacts on the EEG recordings. We found reduced number of gait cycles correlated to an average template for the independent components extracted from the blindfolded condition (Figure 8). In addition, we found slightly reduced time-locked spectral fluctuations during blindfolded walking for the cluster of motion-related Independent components, as well as reduced influence of EEG cleaning using these independent components on the alpha absolute power of EEG channels. These results indicate TCR can be sensitive to slight changes in head dynamics caused during different motion conditions, but more research involving different walking speeds and other type of cyclical tasks are needed to further consolidate/validate the usability of this method. Moreover, proper validation procedures for this type of method requires a ground truth measure, which is not possible to extract from scalp EEG during tasks not locked to external events. Recent studies have been exploring phantom heads to establish ground truth measurement during head motion (Chowdhury et al., 2014; Oliveira et al., 2016a). These authors have successfully described the influence of motion-related artifacts on scalp EEG data. Studies using phantom heads are relevant, but the use of a limited number of artificial EEG sources might oversimplify the real conditions of volume conduction and mixed sources encountered during experimental EEG recordings. Nonetheless, future studies using phantom heads may be highly relevant to compare the effectiveness of different methods dedicated to remove/minimize artifacts from EEG recorded during walking.

In summary, in this study we proposed the TCR as a novel method for identifying and rejecting EEG channels and components carrying motion-related artifacts. The method used a template correlation basis and was complemented by subject-specific amplitude breaking point for defining channels to be rejected. Identification of independent components required additional evaluation of scalp topography and spectral power as has been indicated previously (Snyder et al., 2015). The template correlation rejection identified channels and components carrying motion-related artifacts, and their exclusion may reduce the likelihood of mixing artifacts into the neural independent components. We recommend the application of this method for EEG datasets recorded during human locomotion using high-density EEG systems, as larger numbers of channels optimize the identification of amplitude breaking points and provides high resolution of neural content.

## Author contributions

AO, BS, WH, PK, and DF designed the experiments. AO and BS performed data collection. AO, BS, WH, PK, and DF performed data analysis and interpretation of the results. AO and BS drafted the first version of the manuscript. WH, PK, and DF revised the manuscript. AO, BS, WH, PK, and DF read and approved the final version of the manuscript.

## Funding

This work was supported in part by the US Army Research Laboratory ARL-CTA (W911NF-09-1-0139 & W911NF-10-2-0022).

### Conflict of interest statement

The authors declare that the research was conducted in the absence of any commercial or financial relationships that could be construed as a potential conflict of interest.
